# Modified Technique for Retrograde Cerebral Perfusion during Hemiarch Aortic Replacement

**DOI:** 10.1055/s-0041-1726279

**Published:** 2021-10-12

**Authors:** Nicholas T. Kouchoukos, Marc Haynes, Sarah Hester, Catherine F. Castner

**Affiliations:** 1 Division of Cardiovascular and Thoracic Surgery, Missouri Baptist Medical Center, BJC Healthcare, St. Louis, Missouri

**Keywords:** brain protection, hemiarch replacement, retrograde cerebral perfusion

## Abstract

**Background** Uncertainty remains regarding the optimal method of brain protection for procedures that require repair or replacement of the aortic arch. We examined the early outcomes of a technique for brain protection in patients undergoing partial aortic arch (hemiarch) replacement that involves deep hypothermic circulatory arrest (DHCA) and retrograde cerebral perfusion (RCP) of cold blood from the superior vena cava toward the end of the arrest interval.

**Methods** During a recent 15-year interval, 520 patients underwent elective or urgent/emergent ascending aortic and hemiarch replacement as an isolated (47 patients) or combined (473 patients) procedure employing DHCA (mean nasopharyngeal temperature at circulatory arrest, 17.1°C and mean duration, 19.3 minutes) supplemented with RCP of cold blood from the superior vena cava toward the end of the arrest interval (mean, 6.7 minutes). The mean age of the patients was 59.5 years, and 65% were male.

**Results** The in-hospital and 30-day mortality rates were 1.2% (six patients). Seven patients (1.4%) sustained a stroke and 19 patients (3.7%) had transient neurologic dysfunction that completely resolved by the time of hospital discharge. Four patients (0.77%) developed postoperative renal failure requiring dialysis. Twenty-one patients (4%) required ventilator support for >48 hours and five patients (0.96%) required a tracheostomy. The median hospital length of stay was 6 days.

**Conclusion** DHCA with a brief interval of RCP is a safe and effective technique for brain protection during hemiarch aortic replacement. RCP reduces the duration of brain ischemia and permits removal of particulate matter and air from the arterial circulation.

## Introduction

The optimal method of brain protection for procedures that require repair or replacement of the aortic arch has not been clearly established. For patients who require total aortic arch replacement, often in combination with procedures on the ascending or descending thoracic aorta, there is emerging consensus that some form of brain perfusion is advisable during the interval of circulatory arrest which often exceeds 30 to 40 minutes, to prevent neurologic injury. 1 2 For patients who require partial replacement of the arch that does not involve the origins of the brachiocephalic arteries (hemiarch procedure) and generally involves shorter intervals of brain ischemia, the options for brain protection include deep hypothermic circulatory arrest (DHCA), retrograde cerebral perfusion (RCP) of cold blood into the superior vena cava in combination with DHCA, occlusion of the brachiocephalic arteries and perfusion of the brain through the right axillary or innominate artery, and direct antegrade cerebral perfusion (ACP) of two or all three brachiocephalic arteries. The latter two techniques have been used in combination with deep or moderate hypothermia.

In 1994, we described a technique for brain protection during operations involving the aortic arch that utilizes DHCA and retrograde perfusion of the brain with cold, oxygenated blood from the superior vena caval cannula near the end of the interval of HCA as an adjunctive measure for brain protection. 3 In this report, we present our experience with this technique in patients who underwent hemiarch replacement over a recent 15-year interval.

## Materials and Methods

Between January 2001 and December 2015, 520 patients underwent elective or urgent/emergent ascending aortic and hemiarch replacement as an isolated or combined procedure employing an interval of DHCA supplemented with retrograde brain perfusion of cold blood from the superior vena cava toward the end of the arrest interval. The rationale for the retrograde blood perfusion was to reduce the interval of brain ischemia and to remove particulate matter (thrombus, calcium, and atheromatous debris) and air from the arterial circulation. 3 During the study interval, no other technique for brain protection was utilized for patients undergoing hemiarch replacement. The mean age of the patients was 59.5 ± 13.8 years and 338 (65%) were men. One hundred and forty-eight patients (28.5%) were 70 years of age or older, and 23 patients (4.4%) were 80 years of age or older. Data were analyzed retrospectively from a prospectively maintained database. Thirty-day follow-up was available for all 520 patients. 4 The study was reviewed by the Institutional Review Board of the Missouri Baptist Medical Center and was exempted from board approval.

The principal indications for operation are shown in Table 1 . Four hundred and nineteen patients (80.6%) had either aortic regurgitation or aortic stenosis as the indication for operation and had associated aneurysmal enlargement of the ascending aorta and proximal aortic arch. Ascending aortic and proximal aortic arch enlargement was the indication in 74 patients, 11 patients had severe atherosclerosis of the aortic segment, 7 patients had acute Type A aortic dissection, and 9 patients had chronic Type A aortic dissection with aneurysm formation. One hundred and two patients (19.6%) had undergone a previous cardiac or ascending aortic procedure and 11 patients (2.1%) had undergone two or more procedures. The procedures performed on the aortic valve are shown in Table 2 . Additional procedures that were performed concomitantly in 178 patients (34.3%) are shown in Table 3 . Fifty-two of the 101 patients with aneurysm or other ascending aortic disease as the principal indication for operation required an additional procedure.

**Table 1:** Principal indications for operation

Indication	No. of patients	%
*Aortic valve disease* :		
Regurgitation	286	55.0
Stenosis	133	25.6
*Ascending aortic and proximal aortic arch disease* :		
Aneurysm	74	14.2
Atherosclerosis	11	2.1
Acute dissection	7	1.4
Chronic dissection	9	1.7
Total	520	100

**Table 2:** Procedures on the aortic valve a

Condition	No. of patients	%
*Aortic root replacement* :	202	45.9
Mechanical valve + graft	114	
* Bioprosthetic valve* :		
Stentless root graft	83	
Valve + graft	5	
*Aortic valve* :	144	32.7
Pericardial valve	94	
Mechanical valve	50	
*Valve sparing procedure* :	61	13.8
Commissural aortoplasty	26	
David procedure	24	
Yacoub's procedure	7	
Leaflet repair	4	
Allograft root replacement	20	4.6
Ross procedure	13	3.0
Total	440	100

a 21 patients with aneurysm as the principal indication for operation had a procedure on the aortic valve or the aortic root.

**Table 3:** Additional procedures in 178 patients a

Procedure	No. of procedures	%
*Coronary artery bypass grafting* :	116	54.0
One	49	
Two	44	
Three or more	23	
*Polyester grafts to coronary ostia* :	28	13.0
Left main coronary	17	
Right coronary	1	
Both	10	
Mitral valve repair/replacement	20	9.3
*Maze procedure* :	10	4.7
Left only	7	
Bilateral	3	
Tricuspid valve repair	5	2.3
*Other* :	36	16.7
LVOT reconstruction	15	
Closure patent foramen ovale	9	
Ventricular septal myectomy	4	
Ablation for atrial flutter	2	
Permanent pacemaker	2	
Pulmonary valve replacement	2	
Carotid endarterectomy	2	
Total	215	100

Abbreviation: LVOT, left ventricular outflow tract.

a Several patients had more than one procedure.

### Operative Technique

After a full or partial median sternotomy incision, either the ascending aorta, the right axillary artery or a femoral artery was cannulated ( Table 4 ). The vena cavae were cannulated separately. If cannulation through the right atrial wall was not possible, a long multistage catheter was inserted through a femoral vein with the tip positioned in the superior vena cava. After establishing cardiopulmonary bypass (CPB), cooling was initiated, the left heart was vented, and a cannula was inserted into the coronary sinus for delivery of cold blood cardioplegic solution. During cooling, the head was packed in ice, electroencephalographic and cerebral oxygen saturation monitoring were implemented, and intravenous methylprednisolone (7–10 mg/kg) was administered. After occlusion of the aorta, cardioplegic solution was administered into the coronary sinus at every 12 to 15 minutes. If the duration of aortic occlusion was prolonged to greater than 60 minutes, additional cardioplegic solution was infused directly into the coronary ostia or into grafts that had been attached to the coronary arteries, and topical cooling was added. When the electroencephalogram became isoelectric and the nasopharyngeal temperature reached 18°C or below, the patient was placed in a steep Trendelenburg's position and the superior vena caval cannula was clamped for 8 to 10 seconds to distend the upper venous system and to prevent suctioning of air into the brachiocephalic arteries when the aorta was opened. If a single venous cannula was used, it was occluded for a similar interval. Circulatory arrest was then established.

**Table 4:** Cardiopulmonary perfusion data

Variable	Value
*Arterial cannulation site* :	
Ascending aorta	425
Right axillary artery	62
Femoral artery	33
*Duration (min)* :	
Cardiopulmonary bypass	130.8 ± 43.2
Aortic occlusion	119.4 ± 38.1
Cooling	27.7 ± 6.0
Circulatory arrest	19.3 ± 5.1
*Retrograde brain perfusion* :	
Minutes	6.7 ± 2.4
Rate (mL/min)	473.4 ± 166.8
Jugular venous pressure (mm Hg)	24.1 ± 7.2
*Temperature (°C)* :	
Lowest nasopharyngeal	17.1 ± 1.8
Lowest bladder	22.9 ± 3.0

Note: Data presented as *n* or as mean ± standard deviation.

The aortic clamp and the ascending aortic cannula (if present), were removed. The aorta was opened and transected obliquely beneath the origins of the brachiocephalic arteries which were not clamped or internally occluded. The length of aorta to be excised beneath the brachiocephalic arteries was determined by the extent of aneurysmal enlargement or atherosclerosis and could extend to the origin of the left subclavian artery. The opened aorta was sutured to an appropriately beveled collagen impregnated polyester graft (Hemashield, Maquet, Ratstatt, Germany). As this suture line was being completed, the superior vena cava was occluded with a tourniquet, and cold (18–20°C) arterial blood was infused through the superior vena caval cannula for 5 to 8 minutes at a rate of 300 to 600 mL/min. The jugular venous pressure was not allowed to exceed 33 to 35 mm Hg. If a single venous cannula was used, a balloon-tipped cardioplegia cannula was inserted through a purse-string suture in the superior vena cava above a tourniquet, and the cold blood was infused through this catheter. When the suture line was completed and if a central aortic cannula was used, it was reinserted through a stab wound in the graft beneath the brachiocephalic arteries and secured with a purse-string suture. Air was evacuated from the descending thoracic aorta and the aortic graft by infusion of blood from the pump oxygenator through the aortic cannula or axillary or femoral artery. The aortic graft was occluded with a clamp just proximal to the origin of the innominate artery, CPB was reestablished, and rewarming was initiated. During rewarming, the necessary aortic root, aortic valve, and other procedures were performed. After completion of these procedures and evacuation of air from the heart, CPB was discontinued when the nasopharyngeal temperature reached 35°C. The data related to cardiopulmonary perfusion are summarized in Table 4 .

## Results

### Early Mortality

The in-hospital and 30-day mortality rates were 1.2% (six patients; Table 5 ). Two patients died in the early postoperative period. A 52-year-old male with severe aortic regurgitation and massive cardiomegaly had dense inflammatory changes in the aortic root that precluded attachment of the left main coronary artery to the aortic graft at the time of aortic root replacement. He had placement of a saphenous vein graft from the aortic graft to the left main coronary artery and a subsequent vein graft to the left anterior descending coronary artery but succumbed from biventricular failure on the operative day. A 62-year-old male with critical aortic stenosis, previous mantle irradiation for Hodgkin's lymphoma and coronary artery bypass grafting, had replacement of his severely atherosclerotic aorta, the aortic root, and the mitral valve. He died on the first postoperative day of biventricular failure despite insertion of an intra-aortic balloon pump and a temporary right-ventricular assist device. Three male patients, who were 72, 75, and 78 years of age, died on postoperative days 15, 23, and 36, respectively, of multiple system organ failure. All three had extensive coronary artery disease that required bypass grafting, and two had severe atherosclerosis of the ascending aorta and arch that required graft replacement. The sixth patient, a 76-year-old female died on the 25th postoperative day from ischemic bowel necrosis following repair of an acute Type A aortic dissection. The early mortality rate for the 372 patients under the age of 70 years was 0.5% (two patients), and for the 148 patients who were 70 years of age or older was 2.7% (four patients). The difference was not statistically significant ( *p* = 0.06; Fischer's exact test statistic).

**Table 5:** Postoperative mortality and major morbidity

	No. of patients	%
Early death	6	1.2
Stroke	7	1.4
*Temporary neurologic dysfunction*	19	3.7
Seizures	3	
Delirium	4	
Confusion	12	
Renal dysfunction		
Serum creatinine >1.5 × baseline	23	4.4
Dialysis a	4	0.77
Pulmonary dysfunction		
Ventilatory support >48 hours	21	4.0
Tracheostomy b	5	0.96

a Three patients successfully weaned, one died.

b Four patients successfully weaned, one died.

### Major Morbidity

The prevalence of postoperative neurologic, renal, and pulmonary complications is shown in Table 5 .

#### Neurologic Injury

Seven patients (1.4%) sustained a stroke confirmed by computed tomographic (CT) or magnetic resonance (MR) imaging and by consultation from a neurologist. At the time of discharge, the neurologic deficits had resolved in four of the seven patients. Nineteen patients (3.7%) developed transient neurologic dysfunction ( Table 5 ). Seizures occurred in three of these patients. CT imaging showed no evidence for brain injury in all three patients. The seizures resolved, and none of the patients required antiseizure medication. Delirium occurred in 4 of the 19 patients and CT imaging was negative for brain injury in all 4 of them. The remaining 12 patients had major confusion. CT imaging was obtained in six of these patients and no abnormalities were noted. All of the 19 patients had resolution of their neurologic symptoms by the time of hospital discharge.

#### Renal Dysfunction

Preoperative renal dysfunction, defined as a baseline serum creatinine level >1.5 mL/dL, was present in 10 patients (1.9%). Postoperative hemodialysis was required in two of these patients. One patient died in the early postoperative period and the other patient required only temporary renal replacement therapy. Twenty-three patients (4.4%) with normal renal function preoperatively developed postoperative dysfunction defined as an elevation of the serum creatinine ≥1.5 times the baseline level ( Table 5 ). Two of these patients required hemodialysis that was discontinued prior to hospital discharge.

#### Pulmonary Complications

Twenty-one patients (4%) required ventilator support for more than 48 hours, and five patients (0.96%) required tracheostomy and prolonged assisted ventilation. Four of the five patients were weaned from the ventilator and had removal of the tracheostomy tube before hospital discharge. The fifth patient died in the postoperative period ( Table 5 ).

#### Other Morbidity

Eighteen patients (3.5%) required reoperation for bleeding ( Table 6 ). Delayed closure of the sternum was performed in 47 patients (9%) in the first 24 to 48 hours postoperatively. Twenty-two of these patients had undergone a previous sternotomy. Intra-aortic balloon pumping was required in five patients. New onset atrial fibrillation occurred in 112 patients (21%), and 14 patients (2.7%) received a permanent pacemaker. These and other postoperative complications are summarized in Table 6 . The mean length of hospital stay was 7.5 ± 4.9 days (median, 6 days).

**Table 6:** Other postoperative morbidity

Type of morbidity	No. of patients	%
Reoperation for bleeding	18	3.5
Delayed closure of sternal wound	44	8.5
Intra-aortic balloon pump	5	0.78
New atrial fibrillation	112	21.5
Permanent pacemaker	14	2.7
Delayed drainage of pericardial effusion	20	3.9
Deep sternal wound infection	5	0.98

Data regarding intraoperative transfusion of allogeneic blood products were only available in the database for the 419 patients who were operated on after January, 2004. A total of 175 of these 419 patients (41.7%) received no intraoperative transfusion of blood products. The blood products received by the remaining 244 patients are shown in Table 7 . One patient required transfusion of factor VIIa concentrate.

**Table 7:** Intraoperative transfusion of blood products in 244 patients

Blood product	No. of units	
	Median	Interquartile range
Red blood cells	3	2–5
Platelets	2	2–3
Fresh frozen plasma	2	2–4
Cryoprecipitate	1	1–2

## Discussion

Although there is general agreement that some form of brain perfusion (ACP or RCP) is advisable for patients undergoing total arch replacement, there is no consensus regarding the optimal method for brain protection for operations that require subtotal arch replacement. Kaneko and colleagues 5 compared DHCA alone and combined with antegrade or retrograde brain perfusion in 467 patients undergoing hemiarch replacement. Patients with calcified aortas were excluded. They demonstrated comparable outcomes with respect to early mortality, stroke, transient ischemic attack, renal failure, and reoperation for bleeding among the three groups. Other studies comparing these three modalities for brain protection during aortic arch surgery in which the majority of patients underwent hemiarch replacement, also failed to demonstrate differences in mortality and neurologic injury. 6 7 8 Misfeld and colleagues 9 in a study of 636 patients undergoing aortic arch surgery, 71% of whom had partial arch replacement, reported comparable outcomes with regard to 30-day mortality and the occurrence of permanent and temporary neurologic deficits among patients in whom unilateral ACP, bilateral ACP, RCP, and DHCA were used for brain protection. 9 Only when the two antegrade perfusion groups were combined and compared with the combined RCP and DHCA groups, a significant difference was observed in the rate of stroke (9 vs. 15%, *p* = 0.035). DHCA is the common denominator with all of these techniques, and it continues to be used as the sole form of brain protection in some centers. 10 11 12

Although DHCA with RCP continues to be used on a routine basis for hemiarch procedures by only a minority of aortic surgeons, the outcomes at experienced aortic surgery centers where it is used have been highly satisfactory. 13 14 15 16 The early mortality rate at these four centers ranged from (1.3–4.8%). Some of the variability in early mortality is likely related to differing baseline characteristics of the patients. As an example, the percentage of patients presenting with acute Type A aortic dissection varied from 0 to 25%. The rates of stroke and transient neurologic dysfunction ranged from 1.3 to 3.0 and 3.0 to 7.5%, respectively, and the rate of renal failure requiring dialysis ranged from 0.3 to 2.5%. The rate of reoperation for bleeding ranged from 1.3 to 4.3%. RCP in these studies was delivered continuously during the period of DHCA.

In our study, RCP was delivered only at the end of the interval of DHCA, and our outcomes are comparable to those observed in the series noted above. We continue to use DHCA with RCP routinely for hemiarch procedures because of its demonstrated safety and for its simplicity, since it provides an uncluttered operative field and requires only minimal alterations in the perfusion circuit. 3 We administer RCP only during the final minutes of the interval of DHCA, primarily to remove macro- and microembolic materials and air from the arterial circulation, and also to reduce the duration of brain ischemia. In addition, the availability of RCP provides an additional safety factor if the interval of DHCA has to be extended.

Concerns regarding the ability of RCP to provide meaningful suppression of cerebral metabolism and the detrimental effects of deep hypothermia (primarily increased bleeding) have led many aortic surgeons to employ ACP with moderate hypothermic circulatory arrest (MHCA; ACP + MHCA) during procedures on the aortic arch. 2 Leshnower and colleagues 17 performed a randomized trial comparing ACP + DHCA with RCP + DHCA in 20 patients undergoing hemiarch replacement (10 patients in each group). In addition to assessing the usual outcomes, they added mandatory neurologist-adjudicated preoperative and postoperative assessments and postoperative MR neuroimaging. They demonstrated a higher incidence of brain lesions consistent with acute infarction among the patients managed with ACP + MHCA (100 vs. 45%, *p* = 0.01). The mean number of brain lesions per patient was also significantly higher in the ACP + MHCA group (4 ± 3.5 vs. 1.2 ± 2.1. *p* = 0.01). No other significant differences in outcomes were noted between the two groups. Although the clinical significance of the silent infarcts, which are likely embolic in origin, remains unknown, the study demonstrated a lower prevalence of these lesions with DHCA and RCP.

With regard to the issue of excessive bleeding associated with DHCA, several comparative studies have failed to demonstrate significant differences in the frequency of reoperations for bleeding in patients managed with moderate or deep hypothermia. 13 16 18 19 20 Other factors, including preoperative hemoglobin levels, emergency surgery, and the duration of CPB, appear to be more important determinants of increased bleeding. 18 21 22

Few studies comparing the rates of transfusion of allogeneic blood products in patients managed with deep and moderate hypothermia are available. It should be noted that 42% of the 419 patients in our series with transfusion data available received no blood products. In the large series of Girardi and colleagues, 15 where RCP and DHCA were routinely used for aortic arch procedures, 49.8% of the patients undergoing hemiarch replacement required no transfusions.

## Limitations

Our study was observational in nature and involved retrospective review of prospectively collected data at a single referral-based center. All procedures were performed on a single surgical service and the results may not be generalizable. However, the techniques employed are standardized and easily applicable. All of the patients did not undergo preoperative and postoperative formal neurological evaluation and the prevalence of neurologic injury may have been underestimated.

## Conclusion

RCP + DHCA with RCP delivered at the end of the DHCA interval is a safe and effective technique for brain protection for patients who require hemiarch replacement. Our study supports the findings from other studies utilizing RCP which indicated that RCP is an effective method of brain protection in this setting.
